# Giving callers the option to bypass the telephone waiting line in out-of-hours services: a comparative intervention study

**DOI:** 10.1080/02813432.2019.1569427

**Published:** 2019-02-02

**Authors:** J. F. Ebert, L. Huibers, B. Christensen, F. K. Lippert, M. B. Christensen

**Affiliations:** a Section for General Medical Practice, Department of Public Health, Aarhus University, Aarhus C, Denmark;; b Research Unit for General Practice, Aarhus C, Denmark;; c Emergency Medical Services Copenhagen, University of Copenhagen, Copenhagen, Denmark

**Keywords:** Out-of-hours primary care, health services, accessibility, triage, health literacy

## Abstract

**Objective:** Acute out-of-hours (OOH) healthcare is challenged by potentially long waiting time for callers in acute need of medical aid. OOH callers must usually wait in line, even when contacting for highly urgent or life-threatening conditions. We tested an emergency access button (EAB), which allowed OOH callers to bypass the waiting line if they perceived their health problem as severe. We aimed to investigate EAB use and patient characteristics associated with this use.

**Design:** Comparative intervention study.

**Setting:** OOH services in two major Danish healthcare regions.

**Intervention:** Giving callers the option to bypass the telephone waiting line by introducing an EAB.

**Participants:** OOH service callers contacting during end of October to mid-December 2017.

**Main outcome measures:** Proportions of EAB use, waiting time and background information on participants in two settings differing on organisation structure, waiting time and triage personnel.

**Results:** In total, 97,791 out of 158,784 callers (61.6%) chose to participate. The EAB was used 2905 times out of 97,791 (2.97%, 95%CI 2.86; 3.08). Patient characteristics associated with increased EAB use were male gender, higher age, low education, being retired, and increasing announced estimated waiting time. In one region, immigrants used the EAB more often than native Danish callers.

**Conclusion:** Only about 3% of all callers chose to bypass the waiting line in the OOH service when given the option. This study suggests that the EAB could serve as a new and simple tool to reduce the waiting time for severely ill patients in an OOH service telephone triage setting.

**Key Points**
- Acute out-of-hours healthcare is challenged by overcrowding and increasing demand for services.
- This study shows that only approximately 3% of callers chose to bypass the telephone waiting queue when given the opportunity through an emergency access button.
- An emergency access button may serve as a new tool to help reduce the triage waiting time for severely ill patients in out-of-hours medical facilities.

## Introduction

Various options are available for patients in need of medical assistance outside normal office hours. Many countries have out-of-hours (OOH) services, emergency departments (ED), and emergency medical call centres such as the Emergency Medical Dispatch Centre (EMDC-112), which receive emergency calls through the European emergency number 1-1-2 [1–3]. Although these different entities are intended to target different healthcare needs, their patient populations tend to overlap.

A study investigating the contact patterns in Danish OOH services showed that approximately 5% of all callers estimated their condition to be potentially life threatening [4]. A Danish study on urgency levels assessed by triage professionals in an OOH service call centre found that 2.5% of OOH contacts are admitted directly to hospital by ambulance after triage [5]. In a study from Belgium, 1.3% of calls to an OOH clinic were assessed as highly urgent [6]. Likewise, a study on NHS 111 in the UK showed that 9% of callers were referred directly to ambulance services [7]. Citizens often call the OOH services because of worry [8–10], and callers may experience distress due to long waiting time when contacting because of a perceived acute health problem [11]. A combination of long waiting time, worry, and distress could lead to a low degree of feeling safe in the callers [12]. This may prompt a call to the EMDC-112, which is intended only for perceived life-threatening situations requiring immediate medical response, for instance ambulance dispatch. A recent Danish study showed that approximately 20% of all calls to the EMDC-112 are assessed as not relevant for ambulance care [13]. A grey area seems to exist as some of the contacting patients may suffer from serious illness, which could be worsened by prolonged waiting time and delay of treatment, specifically if these patients have not called the most appropriate healthcare service.

An option to bypass the telephone waiting line could provide support to the citizens in greatest need of immediate help and could promote the feeling of safety in callers in distress. This option to bypass the telephone queue is inspired by a similar option in the Netherlands, but little is known about the extent of use. Thus, we aim to test an Emergency Access Button (EAB) serving as an option to bypass the telephone waiting line at the OOH services in two healthcare regions in Denmark with two different organisational settings, including different ranges of waiting time. Specifically, we aimed to investigate the level of use of the EAB, to compare the use between the two settings, and to study the association between patient characteristics and EAB use.

## Material and methods

### Design and setting

OOH service is an important part of Danish primary care as it is the point of entrance to the healthcare system outside normal office hours, ie between 4 pm and 8 am on weekdays, in weekends, and during holidays [2]. We conducted a comparative intervention study at two OOH services with telephone triage: the General Practitioner Cooperative (GPC) in the Central Denmark Region and the Medical Helpline 1813 (MH-1813) in the Capital Region of Denmark. The two settings have been described in Table 1.

**Table 1.** Overview of the two settings included in the study.

Subject	Capital Region of Denmark	Central Denmark Region
Out-of hours service	Medical Helpline 1813 (MH-1813)	GP-cooperative (GPC)
Description of service	Call center available 24/7. (Data is only collected outside office hours in line with the opening hours of the GPC). Organized by the region and managed by a CEO.	Available on weekends and holidays and from 4 pm to 8 am on weekdays. Run by general practitioners (GP).
Triage professionals	Nurses with special training (80%) and medical doctors with varying specialties or in specialty training.	GPs or medical doctors in final year of GP specialty training.
Triage options	Ambulance care Admittance to a hospital Consultation at emergency department Face-to-face consultations by a doctor employed at a hospital Home visit by a doctor Self care	Ambulance care Admittance to a hospital Consultation at emergency department Face-to-face consultation by a GP Home visit by a GP Self care
Contacts per 100 inhabitants/year^a^	52	54
Remuneration	Payment by the hour	Fee for service
Contacts in 2017^b,^^c^	Clinic consultation 48% Home visit 2% Telephone consultation 50%	Clinic consultation 31% Home visit 10% Telephone consultation 59%
Calls per 100 inhabitant/year to EMDC-112^d^	Approximately 4	Approximately 6
Cost for patient	Free	Free

^a^Source: [12]

^b^Source: [26]

^c^Source: [21]

^d^Source: [13,20].

All citizens calling the OOH services must wait in line for triage, regardless of the health problem. The only other option for immediate help is to call the EMDC-112, which has a median telephone response time of around 5 s.

The two different settings were chosen because they represent two different geographical areas and differ in terms of waiting time (i.e. almost twice as long in the MH-1813 as in the GPC) and the educational background of the triage professionals [12]. We hypothesised that longer waiting time is likely to result in higher EAB use. Both settings included an automated message that instructed callers in the telephone waiting queue to hang up and dial 1-1-2 in case of a life threatening situation. The employees, i.e. GPs in the GPC and nurses/doctors in the MH-1813, are referred to as *triage professionals*.

### Intervention

All callers were informed of the project and given the opportunity to decline participation by pressing “1” when entering the telephone queue. They were informed of an ongoing trial aiming to improve the access to out-of-hours care for the severely ill patients and that personal health information could be retrieved for trial participants. All participants were then informed of the estimated waiting time and were subsequently given the option to press “9” if their condition needed immediate medical advice according to their own assessment. The Danish message on the answering machine corresponded to the following: “*If your condition is so severe that you find it necessary to get through straight away, you may press 9 and get first in line. Otherwise please wait.”* Bypassing the telephone waiting line meant jumping to the front of the digital queue and becoming the next in line to talk to a triage professional.

### Data collection

**Figure 1.**
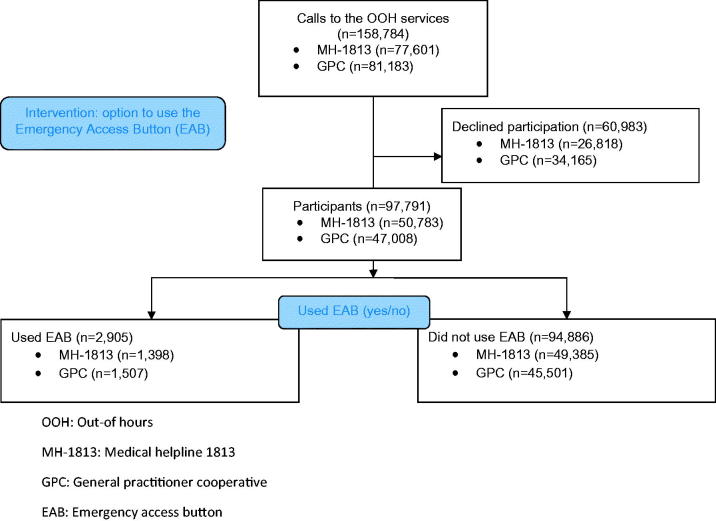
Flow of study participants. OOH: Out-of hours; MH-1813: Medical helpline 1813; GPC: General practitioner cooperative; EAB: Emergency access button.

Data collection for the evaluation of the EAB in two Danish OOH settings started on 27 October 2017 and lasted seven weeks. Callers are routinely asked to enter the civil registration number (CRN) of the patient concerned when calling the OOH services. Approximately 85% of the callers provided this information. Approximately 5% of callers typed in a faulty CRN, which resulted in missing data when we requested socioeconomic data from Statistics Denmark [14]. When no CRN was provided, the triage professional would ask the caller and type it in. The data collection ended on 8 December 2017 for the MH-1813 and on 18 December 2017 for the GPC because more data was needed from the latter setting to ensure sufficient power in our calculations. Exact inclusion and exclusion numbers are shown on the flowchart (Figure 1). Though MH-1813 is open for telephone consultations 24/7, data was only collected during weekends, bank holidays and from 4 p.m. to 8 a.m. on weekdays to match the opening hours of the GPC. The following information was collected for all participants: age, gender, time and date of contact, waiting time (actual waiting time for both settings and announced estimated waiting time for MH-1813), and whether or not they used the EAB. Additionally, socioeconomic data was collected from Statistics Denmark [14]; these included employment status, ethnicity, and highest completed education.

### Analyses

To determine the frequency of EAB use, the two-sample *z*-test of proportions was used. To investigate associations between the rate of EAB use and gender, age group, employment status, educational status, and ethnicity, a logistic regression model was used. To investigate differences in waiting time between the study groups, the Mann-Whitney U test was used. Estimates are presented as interquartile intervals (IQI, p25; p75). We divided the study population into seven age groups based on a similar subdivision in a study by Moth et al. [4]. The subdivision of employment status and education subgroups was inspired by Statistics Denmark [14]. Ethnicity was defined as Danish, western immigrant, or non-western immigrant in accordance with the definition by Statistics Denmark [14]. A citizen was defined as a non-western immigrant if this person or one or both parents had been born outside western Europe, an EU country, Australia, New Zealand or the USA. As we did not gain any information on callers who chose not to participate, we were not able to perform analyses based on non-participants. However, we were able to explore the time and date when a caller had chosen not to participate (data not shown). Analyses were performed in Stata 14 [15].

## Results

Of the 158,784 citizens calling the GPC and the MH-1813, 97,791 (61.6%) chose to participate in the study (Figure 1). Participation was higher in the MH-1813 than in the GPC (65.4% vs. 57.9%). We found that 2905 out of 97,791 (2.97%) callers chose to bypass the telephone waiting line (Table 2). Slightly but significantly fewer patients chose to bypass the queue in the MH-1813 than in the GPC (2.75% vs. 3.21%, diff. 0.45%, CI: 0.24–0.67, *p* < .001). The age group of 0–4 years accounted for 17,777 out of 97,791 (18.7%) contacts, while the age group 61–75 years accounted for only 10,346 out of 97,791 (10.9%) contacts. However, an EAB-use ratio of 327 out of 17,777 (1.84%) contacts for children aged 0–4 years versus 510 out of 10,346 (4.93%) for adults aged 61–75 years indicates that the individuals in the older age group were more frequent users of the EAB. This is supported by the adjusted regression model in Table 3, which shows a trend of increasing EAB use with increasing age. The adjusted odds ratio (OR) for “retired” callers (OR 2.13) is statistically significantly higher than for “employed” callers (OR 1.00). The median actual waiting time was lower in all subgroups in the GPC than in the MH-1813. Furthermore, both settings presented a lower actual waiting time for the citizens who used the EAB compared to the citizens who did not use the EAB (difference in the MH-1813: 45 s for EAB use vs. 177 s for no EAB use). In the MH-1813, EAB users were informed of an estimated waiting time that was significantly higher than callers who did not use the EAB (see Table 2). This is supported by the adjusted regression model, which is presented in Table 3. The model shows that the OR of EAB use increases with longer estimated waiting time.

**Table 2.** Background information on participants and use of EAB stratified for two OOH service settings in Denmark in 2017 (*N* = 97,791).

Setting			GPC			MH-1813			Combined		
						Option to bypass queue					
		Subgroups	All	No bypass	Bypass	All	No bypass	Bypass	All	No bypass	Bypass
Subject			*n =* 47,008	*n =* 45,501	*n =* 1507	*n =* 50,783	*n =* 49,385	*n =* 1398	*n =* 97,791	*n =* 94,886	*n =* 2905
Percentage of bypass			–	–	3.21%	–	–	2.75%			2.97%
	95% CI	–	–	–	(3.05;3.37)	–	–	(2.61;2.90)			(2.86;3.08)
Gender (%)		Missings	5.2	5.1	6.6	2.8	2.8	3.8	3.9	3.9	5.2
	Male		45.8	45.6	51.7	44.3	44.2	47.5	45.1	44.9	49.6
	Female		54.2	54.4	48.3	55.7	55.8	52.5	55.0	55.1	50.4
Agegroup (%)		Missings	2.3	2.3	3.0	2.8	2.8	3.8	2.6	2.5	3.4
	0–4 years		17.2	17.4	9.4	20.0	20.2	14.0	18.7	18.9	11.7
	5–13 years		9.2	9.4	4.6	10.4	10.6	6.1	9.9	10.0	5.3
	14–17 years		4.4	4.5	3.1	4.0	4.0	3.1	4.2	4.2	3.1
	18–40 years		29.3	29.4	26.0	30.6	30.7	26.3	29.9	30.1	26.0
	41–60 years		19.0	18.9	21.5	18.6	18.5	20.7	18.8	18.7	21.2
	61–75 years		11.8	11.5	20.4	10.0	9.8	15.8	10.9	10.6	18.2
	>75 years		9.2	9.0	15.0	6.4	6.2	14.1	7.7	7.5	14.6
Ethnicity (%)		Missings	7.8	7.9	6.0	9.0	9.0	9.5	8.4	8.5	7.7
	Danish		87.6	87.6	87.0	82.2	82.5	70.4	84.8	85.0	79.1
	Western immigrant		2.8	2.8	2.2	3.4	3.4	4.8	3.1	3.1	3.4
	Non-western immigrant		9.7	9.6	10.7	14.4	14.1	24.8	12.1	11.9	17.4
Education (%)		Missings	7.7	7.8	7.2	8.8	8.8	11.6	8.3	8.3	9.3
	<10 years		41.4	41.0	50.5	33.4	33.0	46.7	37.4	37.0	48.8
	10–15 years		36.8	36.9	34.2	37.0	37.1	33.5	36.9	37.0	33.9
	>15 years		21.8	22.1	15.3	29.6	29.9	19.8	25.7	26.0	17.3
Job status (%)		Missings	7.9	7.9	7.2	9.5	9.4	11.1	8.7	8.7	9.1
	Employed		39.9	40.5	25.8	47.9	48.4	30.3	43.9	44.5	27.8
	Under education		15.6	15.7	11.5	17.4	17.7	10.1	16.5	16.7	10.8
	Retired		42.3	41.5	61.3	31.7	30.9	57.3	37.0	36.2	59.5
	Unemployed		2.3	2.3	1.5	3.0	3.0	2.3	2.6	2.7	1.9
Waiting time (seconds)		Missings	0	0	0	0	0	0	0	0	0
	Actual, median		68	72	26	166	177	45	104	111	39
	IQI p25;p75		(11;209)	(11;214)	(11;59)	(15;438)	(13;448)	(34;65)	(12;323)	(12;333)	(23;63)
	Estimated, median		–	–	–	120	120	360	–	–	–
	IQI p25-p75		–	–	–	(60;360)	(0;360)	(180;660)	–	–	–

Missing values in percentage of total n, except for “Education” and “Job status” for which two and three of the youngest age groups, respectively, were excluded. Hence, the percentage of missing values is calculated without these age groups.

EAB: Emergency access button; GPC: general practitioner cooperative; MH-1813: medical helpline 1813; CI: confidence interval; IQI: inter quartile interval.

**Table 3.** Adjusted multiple regression of socioeconomic variables related to EAB use in two OOH settings in Denmark in 2017. (*N =* 97,791).

		GPC			MH-1813			Combined			
Subject	Setting	OR	95% CI		OR	95% CI		OR	95% CI		
Gender										
	Male	1.00			1.00			1.00		
	Female	0.72	0.64	0.81	0.81	0.71	0.93	0.77	0.71	0.84
Age group (years)										
	14–17	0.65	0.40	1.04	0.77	0.46	1.30	0.71	0.50	1.00
	18–40	0.90	0.72	1.12	0.66	0.52	0.85	0.78	0.66	0.91
	41–60	1.11	0.91	1.35	0.77	0.62	0.98	0.95	0.82	1.10
	61–75	1.25	1.04	1.51	0.79	0.64	0.98	1.04	0.91	1.20
	>75	1.00			1.00			1.00		
Ethnicity										
	Danish	1.00			1.00			1.00		
	Western immigrant	1.02	0.65	1.58	1.43	1.01	2.01	1.27	0.97	1.66
	Non-western immigrant	0.95	0.76	1.18	1.91	1.61	2.27	1.47	1.29	1.68
Education										
	<10 years	1.00			1.00			1.00		
	10–15 years	0.80	0.70	0.92	0.68	0.58	0.79	0.76	0.68	0.84
	>15 years	0.67	0.56	0.80	0.53	0.44	0.64	0.61	0.53	0.69
Employment status										
	Employed	1.00			1.00			1.00		
	Student	1.30	1.01	1.66	0.78	0.59	1.04	1.04	0.86	1.25
	Retired	2.02	1.70	2.39	2.44	2.02	2.95	2.12	1.87	2.41
	Unemployed	1.19	0.73	1.94	1.10	0.70	1.72	1.15	0.83	1.60
Waiting time, estimated (minutes)										
	0–2				1.00			1.00		
	3–5				2.43	2.02	2.92	2.41	2.01	2.89
	6–8				2.90	2.35	3.59	2.89	2.34	3.57
	9–11				3.82	3.01	4.85	3.71	2.93	4.70
	12–14				5.45	4.26	6.98	5.33	4.17	6.81
	≥15				6.60	5.25	8.29	2.48	2.15	2.85

Each variable was adjusted for the other variables presented in the table. Estimated waiting time could not be obtained for the GPC. Patients below 14 years of age were exluded because we only had information on the patient in question and not the caller, which is most likely a parent or caregiver. Information on a child’s level of education and employment status would, in this case, not be comparable to the rest of the population.

EAB: Emergency access button; OOH: out-of-hours; GPC: general practitioner cooperative; MH-1813: medical helpline 1813; CI: confidence interval; OR: odds ratio.

The regression model in Table 3 shows the ORs for EAB use adjusted for gender, age group, estimated waiting time (only in MH-1813), ethnicity, education, and employment status. Female gender (OR .77), longer education (>15 years) (OR .61), and decreasing age (OR decreasing from 1 for 75< to .78 for 18-40) were associated with lower EAB use. Ethnicity was not associated with EAB use in the GPC, whereas immigrants tended to use the EAB significantly more than native Danes in the MH-1813.

## Discussion

### Principal findings

We found that 2.97% of patients chose to bypass the telephone waiting line. Slightly but significantly fewer chose to bypass the line in the MH-1813 (2.75%) than in the GPC (3.21%). A considerable fraction of contacts concerned the age group 0–4 years, but the older age groups tended to use the EAB more often. We found a positive association between the announced estimated waiting time and the level of EAB use in the MH-1813. Our adjusted regression model showed that being male, retired, having an education of less than 10 years, and being aged 61–75 years were factors associated with more frequent EAB use. In the MH-1813, being an immigrant also increased the frequency of use.

### Strengths and limitations

We were able to run the intervention for several weeks and to include a considerable number of participants, which gave an accurate estimation of the frequency of EAB use. A great strength was the level of detailed data obtained from the two different settings, including registration of whether or not the EAB had been used, announced estimated waiting times, and actual waiting times. This information was combined with data in the civil registration system through the CRN, which enabled us to collect socioeconomic data and study potential associations between background characteristics and EAB use. However, although we had very complete data on gender, age group, and waiting time, we had two to three times as many missing values in the socioeconomic data. This number of missing values (2–12%) could be explained by faulty entering of CRN digits by the patient, which would imply that the staff at Statistics Denmark [14] was unable to provide us with data on these subjects, or it could be explained by unobtainable information on education and employment status for immigrants.

A limitation of our study was that approximately 38% of callers chose not to participate in the study. The callers declined invitation after being presented with information on the study and the potential retrieval of health records for study participants. The greatest proportion of declined invitations came in the first two opening hours (4 pm–6 pm) on weekdays, where the reasons for calling tend to be less serious [16]. Thus, if 38% of non-participants did have less urgent health problems, this could have led to an overestimation of EAB use in our study. However, another reason for not participating could be that the reason for calling was so severe that the caller was in panic, too worried to care about a research project, or simply missed the invitation because of distress. In this case, our results could have underestimated the actual EAB use.

### Findings in relation to other studies

To our knowledge, this study is the first to test a fast access pathway in an OOH service setting with telephone triage. Therefore, comparison with other studies on the frequency of use is not possible. However, personal communication with Dutch experts on OOH services suggests that approximately 3% of patients use a comparable option to bypass the telephone waiting line in the Dutch OOH service telephone triage (personal communication with Sabine Verheggen, Quality- and complaints officer in the OOH-PC in Nijmegen, The Netherlands). A study from 2012 conducted in the Central Denmark Region found that 5% of patients calling the GPC estimated their situation as potentially life threatening [4] and that 2.5% of all callers were directly referred to the hospital [5]. In a study from Norway from 2009, 2.3% of calls to OOH services were classified as a “red response” that needed immediate ambulance or helicopter dispatch [17]. Similar figures are seen in the Netherlands [18]. Thus, a frequency of use of approximately 3% appears to be a realistic estimate if we assume that only callers with an urgent health problem use the EAB.

We found a difference of .45% between the two studied settings, with lower use in the setting with longest average waiting time. Consequently, we must reject our hypothesis that longer waiting time in one setting would also cause higher frequency of EAB users in this setting. A possible explanation could be that patients accept some waiting time and that the critical limit for acceptable waiting time was not reached during the study. MH-1813 has received quite some media attention on their waiting times being longer than other Danish OOH-services, which could have resulted in a higher threshold for acceptable waiting time among users. Nevertheless, we cannot rule out that settings with longer waiting times could see higher frequencies of use. However, a difference of .45% is not necessarily clinically relevant; it may simply be a significant result owing to high statistical power from many participants. An explanation for the slightly lower frequency in the MH-1813 could be demographic variables. A higher proportion of participants in the MH-1813 belonged to the younger age groups compared to the GPC (Table 2). A trend of more frequent EAB use was seen in the older age groups in both settings, which could be explained partly by a higher frequency of chronic disease among retired citizens (Table 3). However, assigned urgency is known to be lower when the contact concerns a child [4,5,13,19], and this could be part of the explanation for the lower frequency in the MH-1813. A higher proportion of contacts to the EMDC-112 in the Capital Region of Denmark compared to the Central Denmark Region could also partly explain the variation in EAB use between the two settings, i.e. approximately 4 versus 6 per 100 inhabitants each year [13,20]. The extra two contacts per 100 inhabitants account for 36,000 calls in the Capital Region of Denmark, and if these were to call the MH-1813 (approximately 940,000 contacts per year) and use the EAB, this would add approximately .4% to the EAB use and almost cover the difference in use between the two settings [12,13].

The proportion of immigrants was higher in the MH-1813, but it was largely similar to that of the population in the two regions, i.e. 11.2% in the Central Denmark Region and 19.6% in the Capital Region of Denmark [21]. However, we saw a significant association between being an immigrant and using the EAB in the MH-1813. This trend was not seen in the GPC in the Central Denmark Region. Previous studies have reported increased use of OOH services services in areas with high proportions of non-western immigrants [22,23]. Compared to native Danish patients, immigrants tend to perceive higher urgency of their own condition [24,25]. Also, it is possible that some nationalities have a lower threshold for accepted waiting time and have higher expectations to the OOH service than others. This could explain the increased use of the EAB among immigrants in MH-1813.

Even though OOH services instruct callers to hang up and dial 1-1-2 on their telephone waiting lines, both included settings have severely ill patients calling regularly. The EAB could serve as a safety net for those with urgent/severe problems who are stressed and nervous. We can only assume that most of the EAB users were severely ill and that only few non-users were severely ill, to conclude that the EAB ensures short waiting time for severely ill patients. Yet, we cannot rule out that a part of non-users is severely ill.

### Implications for future practice

Optimization and re-organisation of the OOH services are hot topics in Northern Europe, including Denmark. New ways of providing fast high-quality medical advice are constantly tested with the aim to improve the services. The EAB provides an option for OOH service callers to jump the queue in case of perceived severe illness, and it could be an important tool for both service providers and citizens requesting medical attention. The EAB may create a feeling of safety in the citizens because they know that medical advice is quickly accessible in case of severe illness, even at times with long pre-triage waiting time. Our intervention could be implemented at large scale as the level of EAB use seems to be acceptable. Based on results from this study, it has been decided to fully implement the EAB in the MH-1813. Future research should investigate the potential implications of EAB use for the callers. Moreover, the relevance of use should be assessed by triage professionals from a medical perspective.

## Data sharing

The authors are not authorised to share non-aggregated data with a third party. However, data are available from JFE on reasonable request.
